# A new cochliodont anterior tooth plate from the Mississippian of Alabama (USA) having implications for the origin of tooth plates from tooth files

**DOI:** 10.1186/s40851-018-0097-8

**Published:** 2018-06-06

**Authors:** Wayne M. Itano, Lance L. Lambert

**Affiliations:** 1 Museum of Natural History, University of Colorado, Boulder, CO 80309 USA; 2 University of Texas at San Antonio, San Antonio, TX 78249 USA

**Keywords:** Chondrichthyes, Cochliodontiformes, Carboniferous, Mississippian, Bangor limestone, Alabama, Conodonts

## Abstract

**Background:**

Paleozoic holocephalian tooth plates are rarely found articulated in their original positions. When they are found isolated, it is difficult to associate the small, anterior tooth plates with the larger, more posterior ones. Tooth plates are presumed to have evolved from fusion of tooth files. However, there is little fossil evidence for this hypothesis.

**Results:**

We report a tooth plate having nearly perfect bilateral symmetry from the Mississippian (Chesterian Stage) Bangor Limestone of Franklin County, Alabama, USA. The high degree of symmetry suggests that it may have occupied a symphyseal or parasymphyseal position. The tooth plate resembles *Deltodopsis*? *bialveatus* St. John and Worthen, 1883, but differs in having a sharp ridge with multiple cusps arranged along the occlusal surface of the presumed labiolingual axis, rather than a relatively smooth occlusal surface. The multicusped shape is suggestive of a fused tooth file. The middle to latest Chesterian (Serpukhovian) age is determined by conodonts found in the same bed.

**Conclusion:**

The new tooth plate is interpreted as an anterior tooth plate of a chondrichthyan fish. It is referred to *Arcuodus multicuspidatus* Itano and Lambert, gen. et sp. nov. *Deltodopsis*? *bialveatus* is also referred to *Arcuodus*.

## Background

Extant chondrichthyan fishes comprise two clades: the elasmobranchs (sharks, skates, and rays) and the holocephalians (chimaeras). Extant holocephalians possess a dentition consisting of three pairs of tooth plates, a large pair in the mandible and two pairs in the palate [1]. The elasmobranch arrangement of teeth, consisting of rows of tooth files, is thought to be plesiomorphic for crown-group chondrichthyans. Some early holocephalians, e.g., *Helodus*, had a dentition consisting partially of tooth files [2].

Recently, tooth pattern formation in extant elasmobranchs has been studied in great detail, including studies of its embryonic development and gene expression [3–5]. Paleontological studies show that the elasmobranch dental pattern of rows of tooth files, with teeth replaced in a linguo-labial sequence has been highly conserved, since it appears in the early stem-chondrichthyan *Doliodus problematicus* (Emsian, Early Devonian, about 397 Ma) [6]. Comparatively little investigation of tooth plate development in extant holocephalians has been made to date.

How and when the transition from tooth files to tooth plates in holocephalians took place is poorly understood. The age of the most recent common ancestor of modern elasmobranchs and holocephalians has been estimated, by a molecular clock method based on mitogenomic sequences, to be late Silurian, about 421 Ma [7]. Fossil evidence places the date of divergence between the two clades to be no later than the late Carboniferous (Pennsylvanian) [8] or the latest Devonian [9]. Due to the poor fossil record of holocephalians following the end-Permian extinctions, it is not known from which group of Paleozoic holocephalians the extant chimaeroids are descended. Recent studies of tooth plates of the extant holocephalian *Callorhinchus milii* have found that each tooth plate is of a compound nature, representing the fusion of two teeth from a reduced tooth file [1]. Studies of the transition from tooth files to tooth plates are of interest in their own right. They also have the potential of helping to elucidate the phylogeny of the extant holocephalians, particularly when combined with the study of the ontogeny of tooth plates of extant holocephalians.

A holocephalian tooth plate of unusual morphology was recently found in the late Mississippian (early Carboniferous) Bangor Limestone of northern Alabama, USA. An abstract has been published previously [10]. Chondrichthyan remains from the Mississippian of northern Alabama have been reviewed recently [11]. The holocephalian taxa reported from the Bangor Limestone are: *Deltodus* sp. cf. *D. undulatus, Helodus crenulatis,* and *Psammodus* sp.

Fossil tooth plates of holocephalian chondrichthyan fishes are usually found isolated from each other and from other remains. Isolated tooth plates have been classified into species, genera, families, and higher taxonomic categories based on morphology, but their true phylogenetic relationships are often uncertain. Undoubtedly, tooth plates from different positions within the dentitions of the same fishes have been given distinct specific or even generic names. As closely associated or articulated remains are found, some of these genera or species will become junior synonyms of others. There seems to be no reasonable alternative to this somewhat awkward procedure, but it has worked well over time in a similar manner with the multi-element feeding apparatuses of conodonts. The most complete guide to holocephalian fishes is the monograph of Stahl [2]. Only a small fraction of the species listed in that monograph are known from articulated or associated remains.

Among the rare articulated and associated remains are the following: (1) Largely complete single-jaw dentitions of the Mississippian cochliodontiform fishes *Cochliodus contortus* and *Streblodus oblongus* have been reported [12]. It has been proposed that they represent the mandibular and palatal dentitions, respectively, of the same species [12]. However, the association has not been verified directly. (2) Three pairs of tooth plates, from the Pennsylvanian of Ohio, USA, apparently belonging to the same fish, have been found in close association [13]. The two large plates presumed to be mandibular were previously described as *Deltodus angularis,* the two large plates presumed to be palatal, as *Sandalodus carbonarius,* and two small plates presumed to be anterior mandibular, as *Orthopleurodus carbonarius*.

**Fig. 1:**
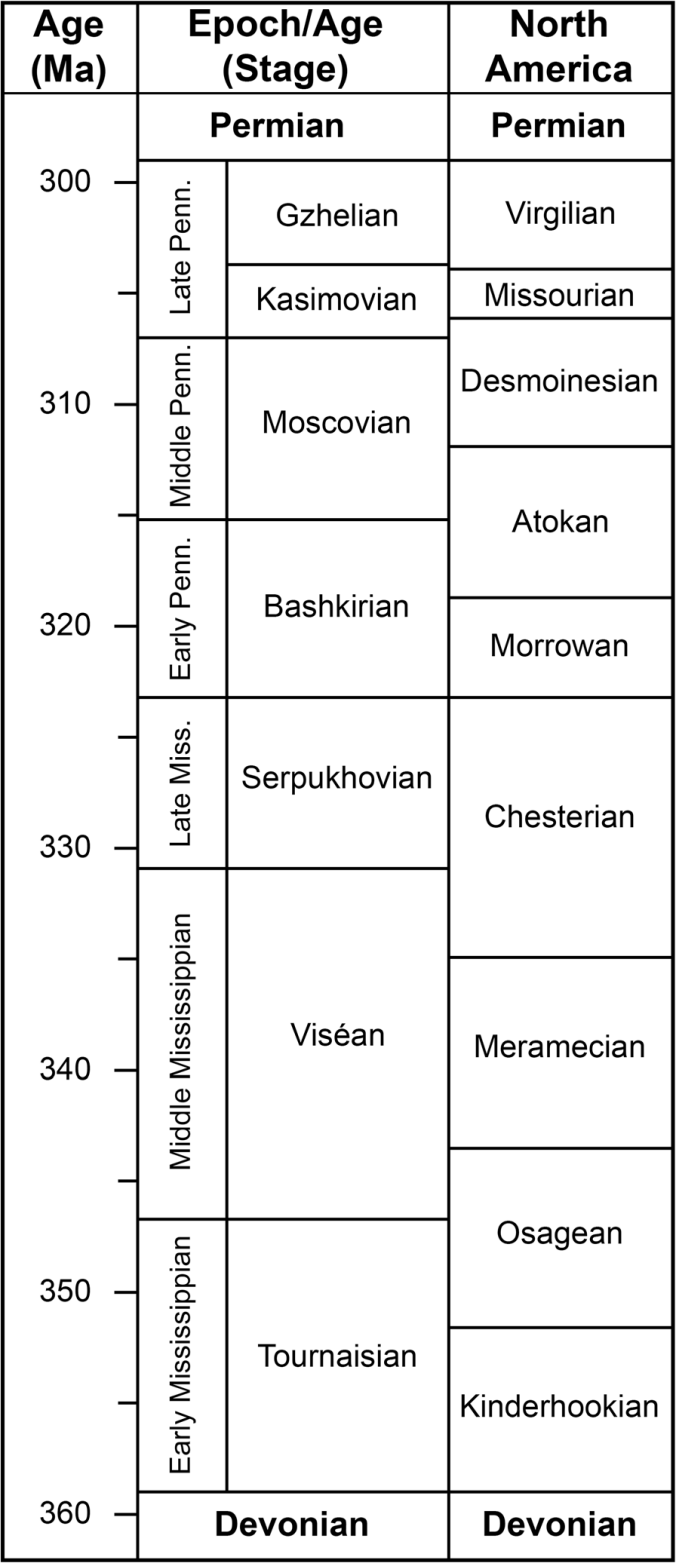
Correlation of standard subdivisions of the Carboniferous Period with North American regional subdivisions. After ([15], fig. 23.1)

Anterior tooth plates of holocephalians are even less well understood than the posterior ones. Being small, they are less likely to be preserved or collected. Apparently none of the articulated *Cochliodus contortus* or *Streblodus oblongus* dentitions preserve the teeth or tooth plates of the extreme anterior region. One of the few holocephalian dentitions preserved with all of the anterior tooth plates in their original positions is that of *Harpagofututor volsellorhinus,* from the Chesterian (Serpukhovian) Bear Gulch Limestone of Montana, USA [14]. Fig. 1 shows thecorrelation between the standard subdivisions of the Carboniferous Period (e.g., Serpukhovian) and the North American regional subdivisions (e.g., Chesterian). The data are taken from ([15], fig. 23.1). *H. volsellorhinus* has three anterior tooth plates (one symphyseal and two parasymphyseal) in the lower jaw and two (parasymphyseal) in the upper jaw. The dentition of another Mississippian holocephalian, *Chondrenchelys problematica* (Order Chondrenchelyformes), has been described recently [16, 17]. In addition to possessing sets of tooth plates in the upper and lower jaws, not too dissimilar to those of Mesozoic and Cenozoic holocephalians, *C. problematica* possesses sets of extramandibular teeth arranged around the periphery of the anterior end of the mouth. This condition is not known in any other chondrichthyan. If found isolated, these anterior teeth would have been identified as those of petalodonts (Order Petalodoniformes). This example illustrates the difficulty of determining whether isolated teeth or tooth plates belong to the same species, as well as the difficulty of identifying isolated chondrichthyan teeth or tooth plates, even to the level of order.

**Fig. 2:**
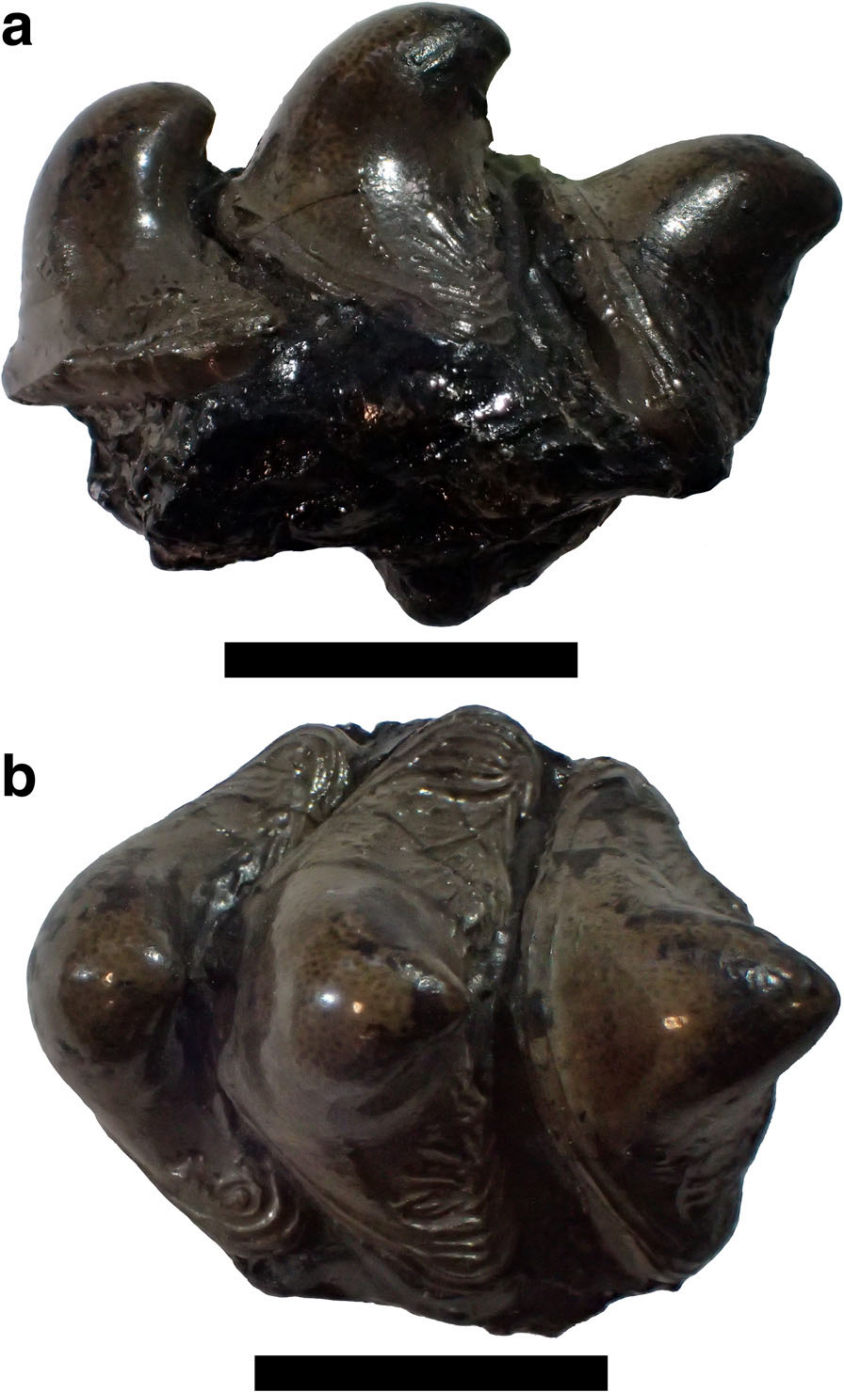
Tooth file of *Helodus simplex* Agassiz, 1838 [18]. One of several specimens labeled NHMUK PV P8216. **a** Lateral view. **b** occlusal view. Scale bars = 1 cm

**Fig. 3:**
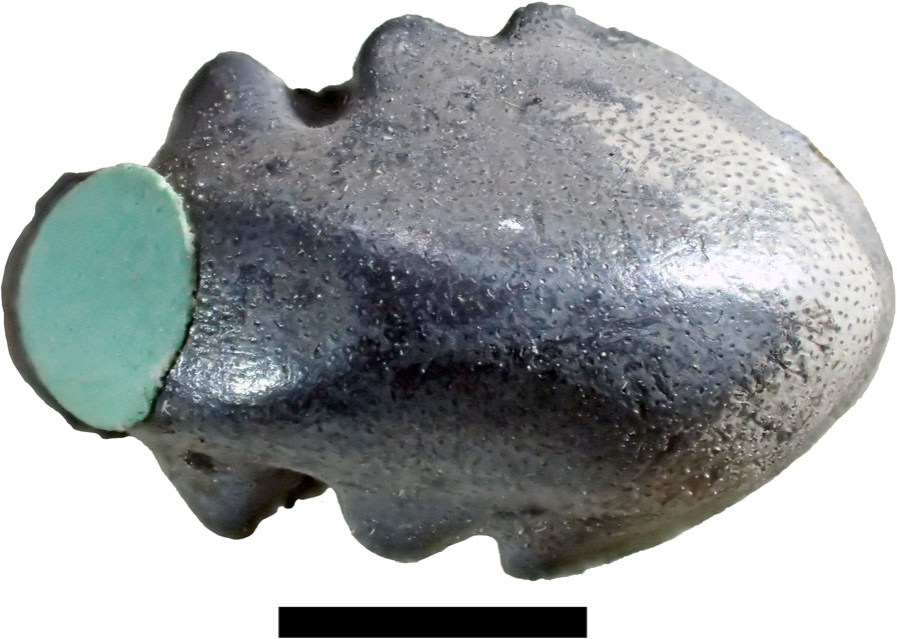
Occlusal view of tooth plate of *Pleuroplax rankinei* (Hancock and Atthey, 1872) [20]*.* One of eight tooth plates labeled NHMUK PV P1415. Lingual end to right. Scale bar = 5 mm

Holocephalian tooth plates are presumed to have evolved by fusion of tooth files. However, there is little fossil evidence for this hypothesis. The dentition of *Helodus simplex* Agassiz, 1838 [18], the type species of *Helodus*, includes both tooth files and tooth plates [19]. Figure 2 shows a tooth file of *Helodus simplex.* The tooth plates in the dentition of *Helodus* have corrugated outlines, which may be remnants of their origin from separate teeth. Isolated tooth plates of this form have been given the genus name *Pleuroplax.* Figure 3 shows a tooth plate of *Pleuroplax rankinei.* Both *H. simplex* and *P. rankinei* are known from articulated remains [19, 20]. These remains show that the two species have a close relationship, but the dentition of *P. rankinei* appears to consist entirely of tooth plates, while that of *H. simplex* includes both tooth files and tooth plates. The anterior tooth plates of *H. volsellorhinus* display longitudinal ridges with bumps that “distinctly resemble fused teeth” [14].

The morphology of the recently-found tooth plate from the Bangor Limestone suggests that it could shed some light on (1) the nature of anterior holocephalian dentitions and (2) on the transition from tooth files to tooth plates.

### Locality

**Fig. 4:**
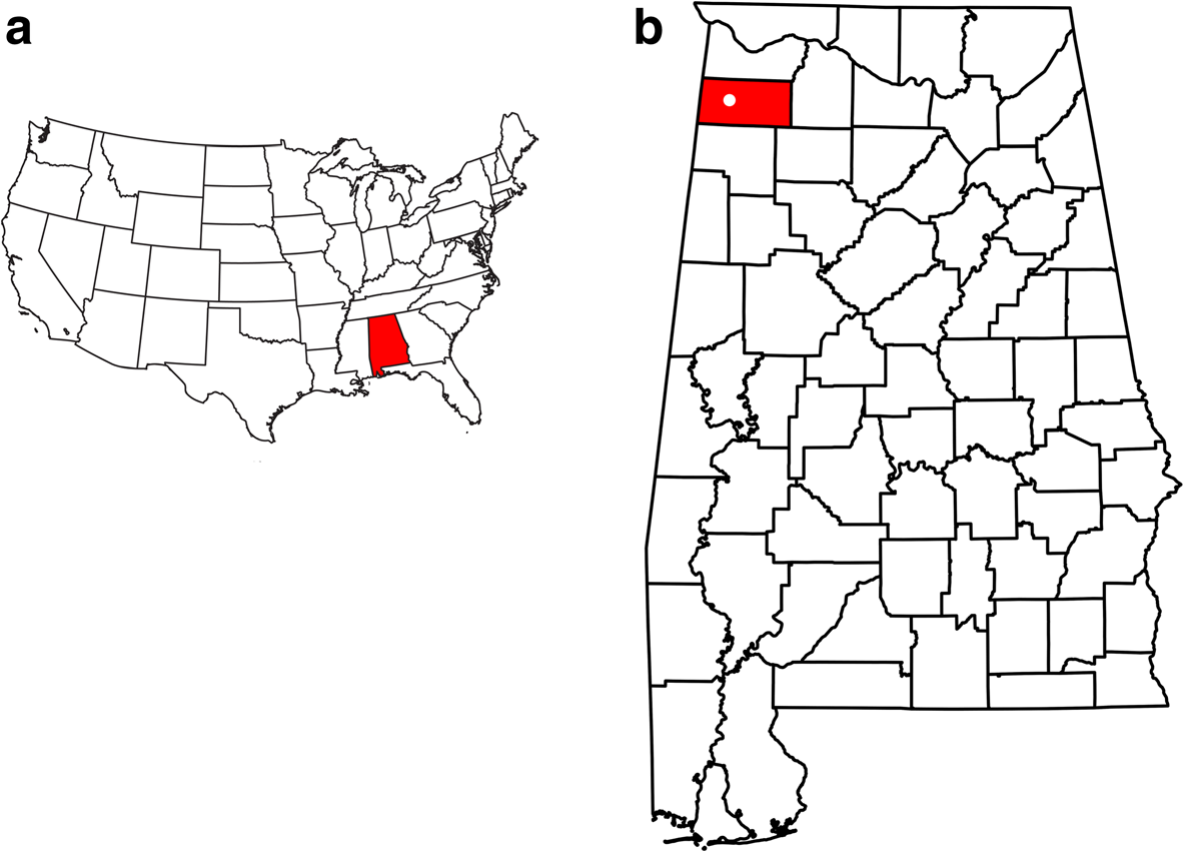
**a** Map of the United States, with the state of Alabama outlined. **b** Map of Alabama with Franklin County highlighted. White dot marks location where holotype of *Arcuodus multicuspidatus* was found

The tooth plate, ALMNH PV 2016.0002.0002, was found in a bed of limestone, near the shore of Little Bear Creek Reservoir, Franklin County, Alabama, USA (Fig. 4). The precise location is on file at the ALMNH and is available to qualified researchers.

## Methods

The piece of matrix containing the tooth plate was immersed in a 5% solution of acetic acid. After 2 days, the fossil was released from the matrix. The acetic acid solution was changed several times during the 2 days. No further preparation was necessary.

Since biostratigraphically useful macrofossils, such as blastoids or crinoids, were not found in the bed, limestone matrix was processed for conodonts by standard acidization methods (e.g., [21]). Approximately 2 kg of limestone matrix were broken into centimeter-sized pieces and immersed in a 15% solution of formic acid for 24 h. The insoluble residues were then wet-sieved through 850 and 125 μm screens. Once dried, the residue from the 125 μm screen was picked by hand for conodonts using a 000 brush under a binocular microscope.

## Results

### Conodont biostratigraphy

**Fig. 5:**
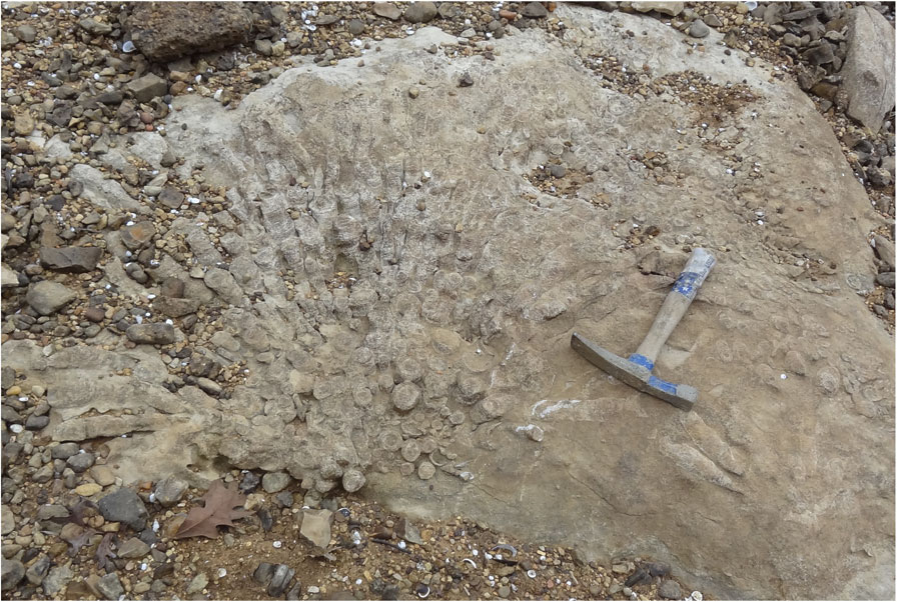
Rugosan coral cluster at the type locality of *Arcuodus multicuspidatus.* The holotype specimen was found, displaced horizontally, at the same stratigraphic level. Rock hammer included for scale. Photograph by L. White. Used with permission

The bed in which the tooth plate was found lies within the Bangor Limestone, which is Chesterian (early Carboniferous = Mississippian) in age [22–25]. The Bangor Limestone in Franklin County is approximately 150 m thick [23]. The bed in which the tooth plate was found is an indurated, medium-gray, bioclastic grainstone. The grains are numerically dominated by crinoid ossicles, which along with abundant bryozoan fragments and a high diversity of other fossil fragment types suggest normal marine salinity. The grainstone fabric and abundant rounded grains indicate high current or wave energy in a shallow marine setting. Laterally in the same bed, the presence of prominent rugosan coral clusters indicates a heterogenous sea floor and a diverse shallow marine environment (Fig. 5).

Conodont P1 elements identified in the matrix from the bed were *Cavusgnathus regularis, C. unicornis* (α), *C. unicornis* (β), and *C. naviculus*. According to [26], *C. naviculus* has the most restricted stratigraphic range of these conodonts. Presence of *C. naviculus* fixes the age of the bed as no earlier than Biozone 2 and no later than Biozone 4 of [26], middle to latest Chesterian North American regional stage. The combined middle and late Chesterian correlates closely with the international standard Serpukhovian Stage, which is late Mississippian (Fig. 1).

### Systematic paleontology

Chondrichthyes Huxley, 1880 [27].

Euchondrocephali Lund and Grogan, 1997 [28].

Holocephali Bonaparte, 1838 [29].

Cochliodontiformes Obruchev, 1953 [30].

### *Arcuodus* Itano and Lambert, gen. nov., urn:lsid:zoobank.org:act:F03B0809-A0DE-475B-9E12-5B3E231A319C

#### Etymology

From Latin arcus = arc and Greek ὀδούς = tooth.

#### Type species

*Arcuodus multicuspidatus* Itano and Lambert, sp. nov.

#### Other included species

*Deltodopsis*? *bialveatus* St. John and Worthen, 1883 [31].

#### Diagnosis

Tooth plates presumed to occupy an anterior position. Elongated labiolingually, compressed laterally. Bilaterally symmetric relative to labiolingual axis or nearly so. Width and height increase lingually. Occlusal surface shows presence of tubular dentine. Smooth parts of lateral surfaces become narrow basally and have concave curvature when viewed from labial or lingual ends. Basal surface smooth and concave.

#### Remarks

**Fig. 6:**
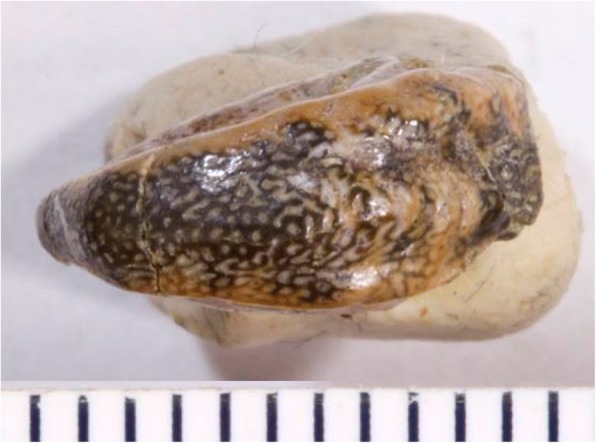
Occlusal view of holotype of *Deltodopsis*? *bialveatus* St. John and Worthen 1883, USNM V13017. Here designated as *Arcuodus bialveatus,* comb. nov. Lingual end to right. Millimeter scale divisions. Drawings of this specimen were published as St. John and Worthen, 1883 ([31], pl. 11, fig. 15). Image copyrighted, Smithsonian Institution, all rights reserved

**Fig. 7:**
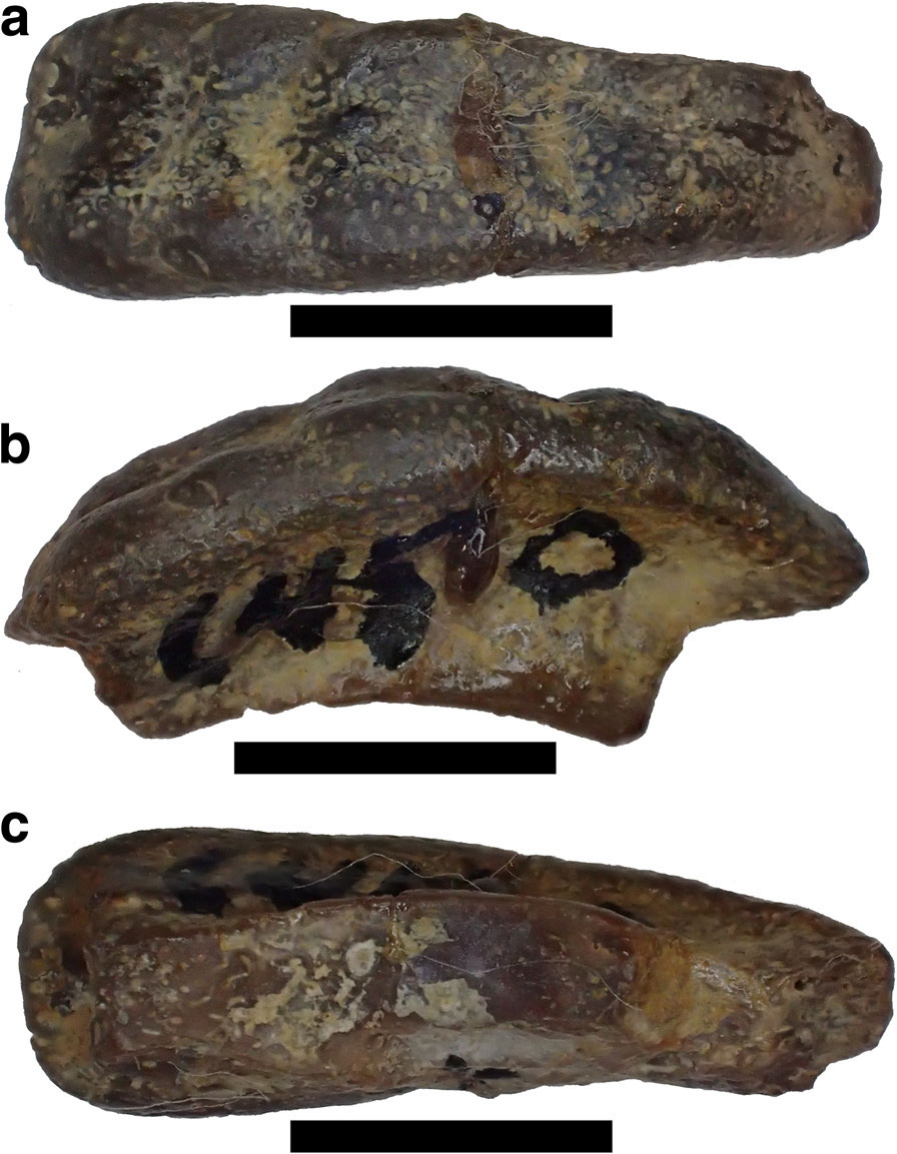
Specimen referred to *Deltodopsis*? *bialveatus*, AMNH FF6450. Here designated as *Arcuodus bialveatus*, comb. nov. **a** Occlusal view. Lingual end to left. Scale bar = 5 mm. **b** Lateral view. Lingual end to left. Scale bar = 5 mm. **c** Basal view. Scale bar = 5 mm. Drawings published as Branson, 1906 ([32], pl. 41, figs. 8-9)

**Fig. 8:**
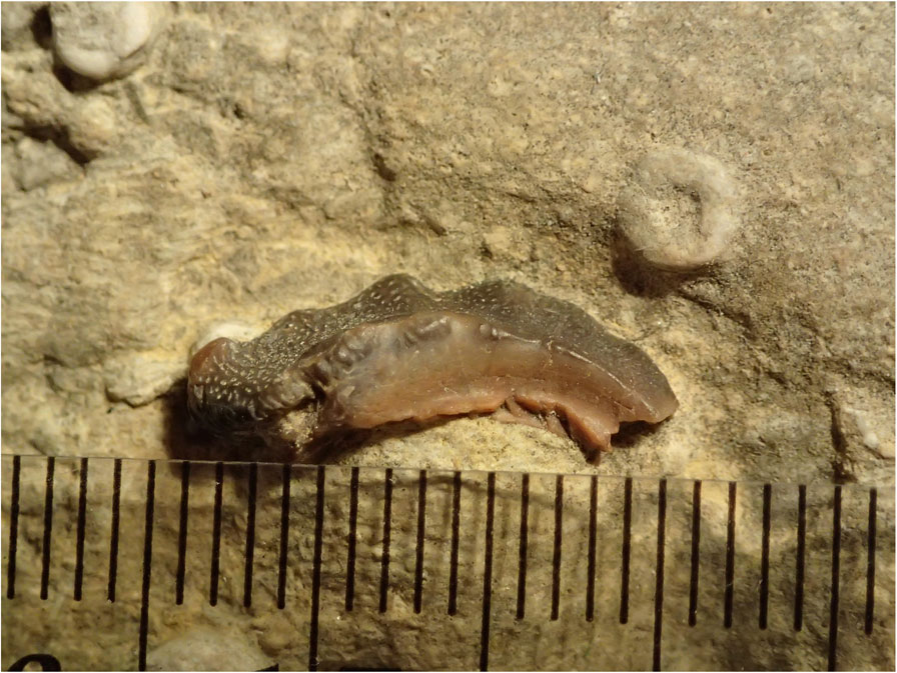
Lateral view of holotype of *Arcuodus multicuspidatus,* Itano and Lambert, gen. et sp. nov., ALMNH PV 2016.0002.0002, prior to extraction from the matrix. Millimeter scale divisions

**Fig. 9:**
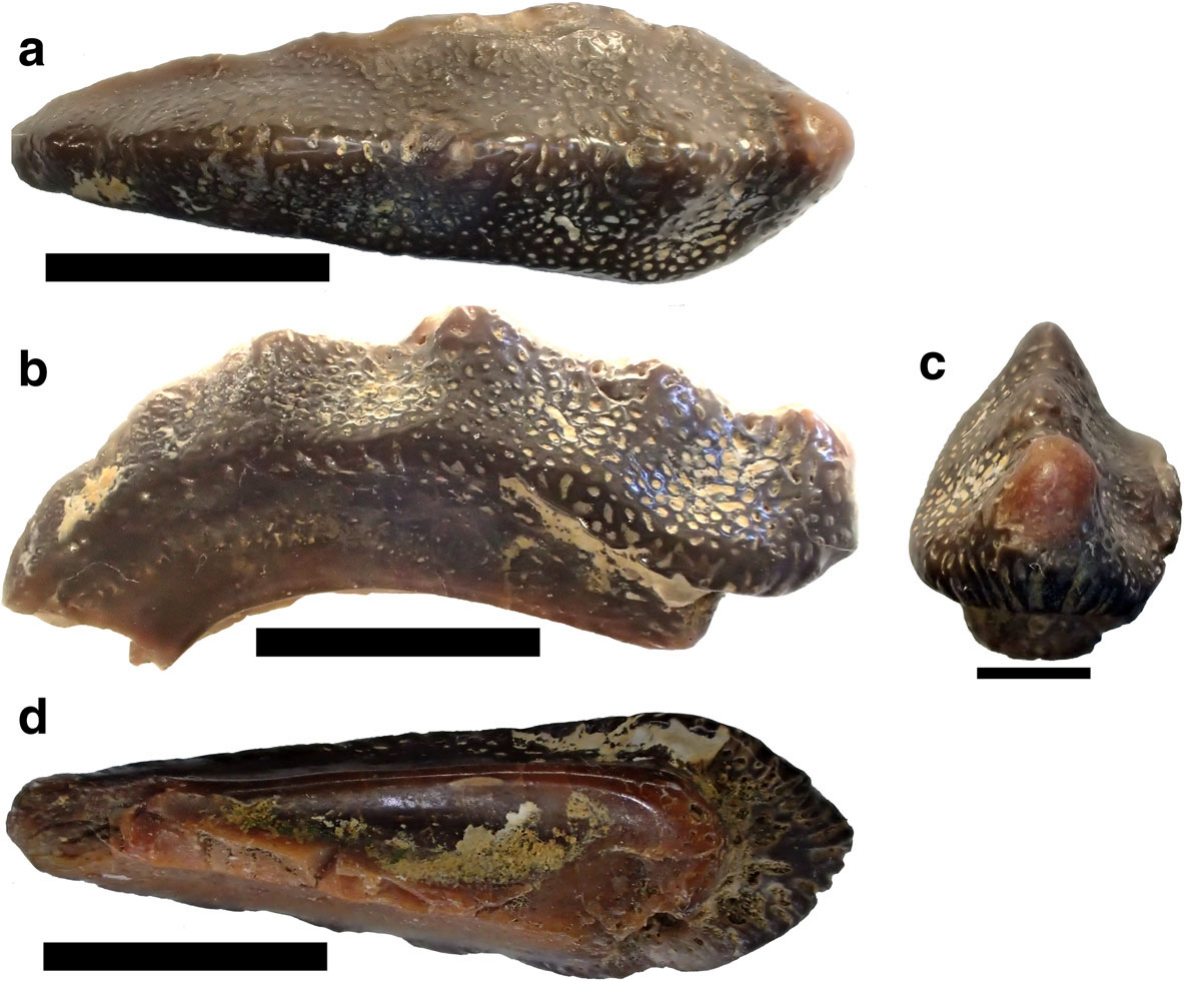
Holotype of *Arcuodus multicuspidatus.* **a** Occlusal view. Lingual end to right. Scale bar = 5 mm. **b** Lateral view. Lingual end to right. Scale bar = 5 mm. **c** Lingual view. Scale bar = 2 mm. **d** Basal view. Lingual end to right. Scale bar = 5 mm

The new genus includes some specimens assigned by St. John and Worthen [31] to their new genus *Deltodopsis,* with some uncertainty, such as *Deltodopsis*? *bialveatus* (Figs. 6 and 7). They did not designate a type species for *Deltodopsis.* However, only the species *D. affinis, D. sanctoludovici,* and *D. angustus* were assigned without question to *Deltodopsis.* All three species are currently assigned to *Deltodus*, according to Stahl [2]. If this assignment is accepted, *Deltodopsis* is a junior synonym of *Deltodus.* Even if the assignment to *Deltodus* is not accepted, a new generic name is required for the new tooth plate (Figs. 8 and 9) and for *Deltodopsis*? *bialveatus,* since they cannot be shown definitively to belong to the same genus as *Deltodopsis affinis, Deltodopsis sanctoludovici,* or *Deltodopsis angustus*. It is likely that the tooth plates referred to *Arcuodus* belong to the anterior parts of the dentitions of fish for which the more posterior tooth plates have already been given generic names, such as *Cochliodus*. If an articulated dentition of such a fish is found, including tooth plates referable to *Arcuodus* and also to a previously named genus, *Arcuodus* would become a junior synonym of the previously named genus.

### *Arcuodus multicuspidatus* Itano and Lambert*,* sp. nov., urn:lsid:zoobank.org:act:AD81711A-3B81-4219-AF6F-924F47B0E1C6

#### Etymology

From the multiple cusps along the occlusal ridge.

#### Type locality

Bangor Limestone, western Franklin County, Alabama, USA; middle to latest Chesterian.

#### Holotype

Tooth plate, ALMNH PV 2016.0002.0002.

#### Diagnosis

Species of *Arcuodus* having tooth plates in which the margin of occlusal surface forms a moderately curved, convex arc with several low cusps. When viewed from lingual or labial ends, occlusal surface appears as a sharply pointed, angular ridge. The multicusped angular ridge distinguishes it from *Arcuodus bialveatus* (St. John and Worthen, 1883)*,* comb. nov., which has a more smoothly convex occlusal surface.

#### Description

The holotype and only known specimen is an isolated tooth plate. The labiolingual length is 15 mm, the width is 4.5 mm, and the height is 5.0 mm. Unknown portions of both the lingual and labial ends are not preserved. The occlusal surface forms a sharp ridge, with six low cusps preserved. The occlusal surface shows presence of tubular dentine. Parallel vascular channels connecting to the surface pores are visible on a broken surface at the lingual end. The outline, seen in occlusal view (Fig. 9a), has a remarkable degree of bilateral symmetry. Some asymmetry can be seen in the labial view (Fig. 9c). Whether this asymmetry is normal or is pathologic is unknown.

### *Arcuodus bialveatus* (St. John and Worthen, 1883) comb. nov.

1883. *Deltodopsis*? *bialveatus;* St. John and Worthen [31], pp. 169–171, pl. 11, fig. 15

1883. *Deltodopsis*? *keokuk;* St. John and Worthen [31], pp. 169–171, pl. 11, fig. 16

1883. *Deltodopsis*? *convexus;* St. John and Worthen [31], pp. 169–171, pl. 11, fig. 17

1906. *Deltodopsis*? *bialveatus;* Branson [32], p. 1391, pl. 41, figs. 8-9

1999. *Deltodus affinis;* Stahl [2], pp. 70–71, fig. 67A

### Holotype

A tooth plate, USNM V13017.

### Referred specimens

Tooth plates, AMNH FF6450, USNM V13015, USNM V13016.

### Occurrence

Burlington Limestone, Louisa County, IA, USA; Keokuk Limestone, Warsaw, IL, USA; Salem Limestone, Lanesville, IN, USA. Mississippian, Osagean to Meramecian = late Tournaisian to Viséan.

### Emended diagnosis

Species of *Arcuodus* having tooth plates in which occlusal surface is more or less smooth and convex, never sharply ridged with cusps as in *Arcuodus multicuspidatus*. Degree of symmetry with regard to the labiolingual axis varies from nearly bilaterally symmetric to moderately asymmetric.

### Remarks

**Fig. 10:**
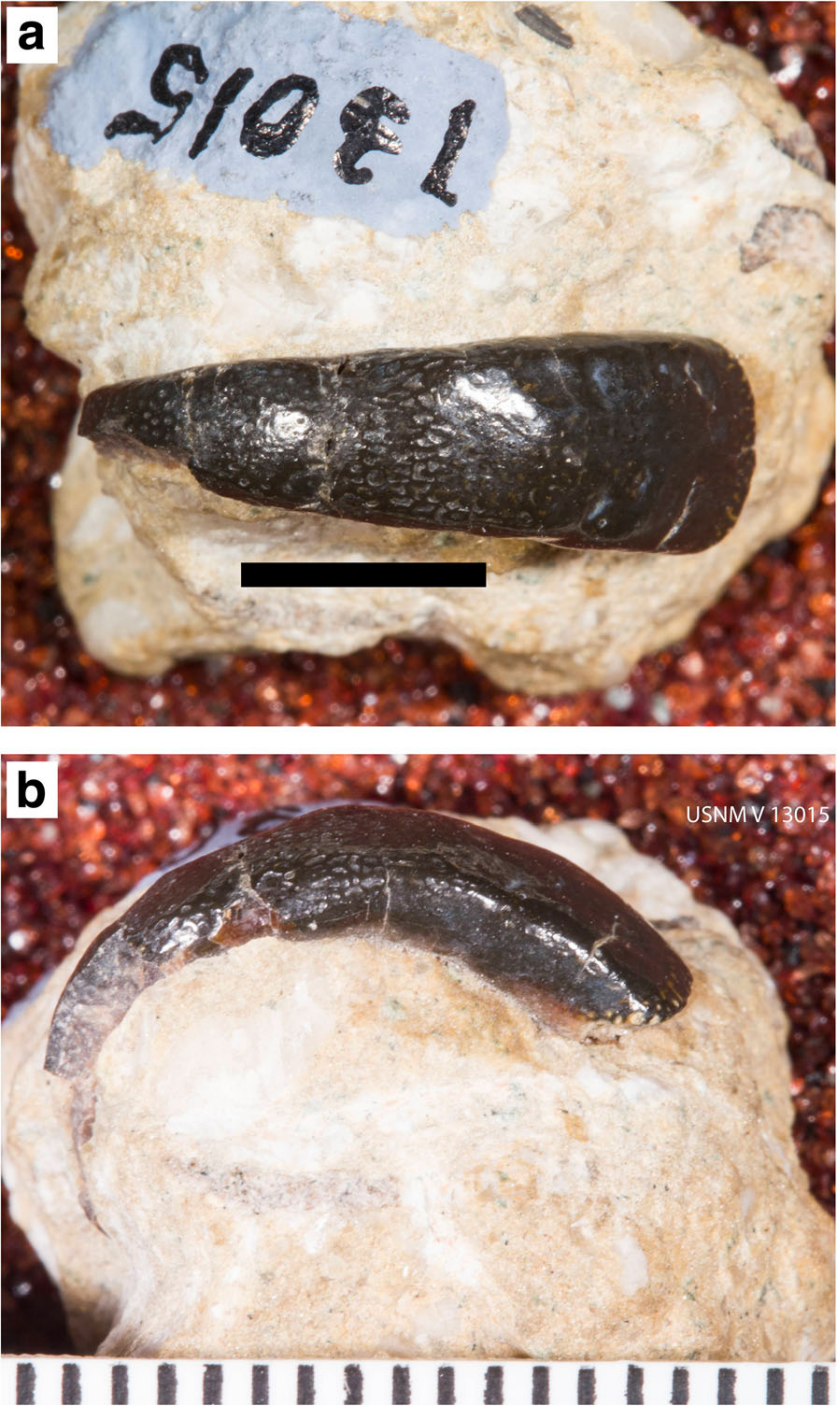
Syntype of *Deltodopsis*? *convexus,* USNM V13015. Here designated as *Arcuodus bialveatus*, comb. nov. **a** Occlusal view. Lingual end to right. Scale bar = 5 mm. **b** Lateral view. Lingual end to right. Millimeters scale divisions. Drawings of this specimen were published as St. John and Worthen, 1883 ([31], pl. 11, fig. 17). Images copyrighted, Smithsonian Institution, all rights reserved

St. John and Worthen [31] defined three species based on small, narrow, tooth plates, referred with some uncertainty to *Deltodopsis,* as *Deltodopsis*? *bialveatus, Deltodopsis*? *keokuk,* and *Deltodopsis*? *convexus.* In defining the three species, based on tooth plates with differing morphologies, they expressed uncertainty as to whether the specimens represented different species or merely varieties. To this uncertainty should be added the positional uncertainty, i.e., whether the tooth plates are mandibular or maxillary and their precise position within either jaw. The high degree of symmetry of USNM V13015 (Fig. 10a) suggests that it may have occupied a symphyseal position. The other specimens are asymmetric, suggesting that they may have occupied a non-symphyseal anterior position. Given the present state of knowledge, the separation of *D.*? *keokuk* and *D.*? *convexus* from *D.*? *bialveatus* would be unjustified. Hence, we refer them both to *Arcuodus bialveatus,* comb. nov. In contrast to the specimens of *D.*? *bialveatus* and *D.*? *convexus* figured by St. John and Worthen [31], the type specimen of *D.*? *keokuk* does not appear to be in the USNM collections. Its whereabouts are currently unknown. On the caption to fig. 67A, Stahl [2] referred the holotype of *Deltodopsis*? *bialveatus,* USNM 13017, to *Deltodus affinis,* but without any justification. Hence, that assignment is not recognized here. The basal structure of *A. bialveatus,* narrowing basally and with a concave basal surface (Figs. 7b–c), is very similar to that of *A. multicuspidatus* (Fig. 9b, d). The occlusal surface of *A. bialveatus,* in contrast to that of *A. multicuspidatus,* lacks sharp cusps, but has shallow undulations which vary from closely spaced (Fig. 7a), to widely spaced (Fig. 10a), to not easily discernable (Fig. 6).

## Discussion

### Anterior dentitions

**Fig. 11:**
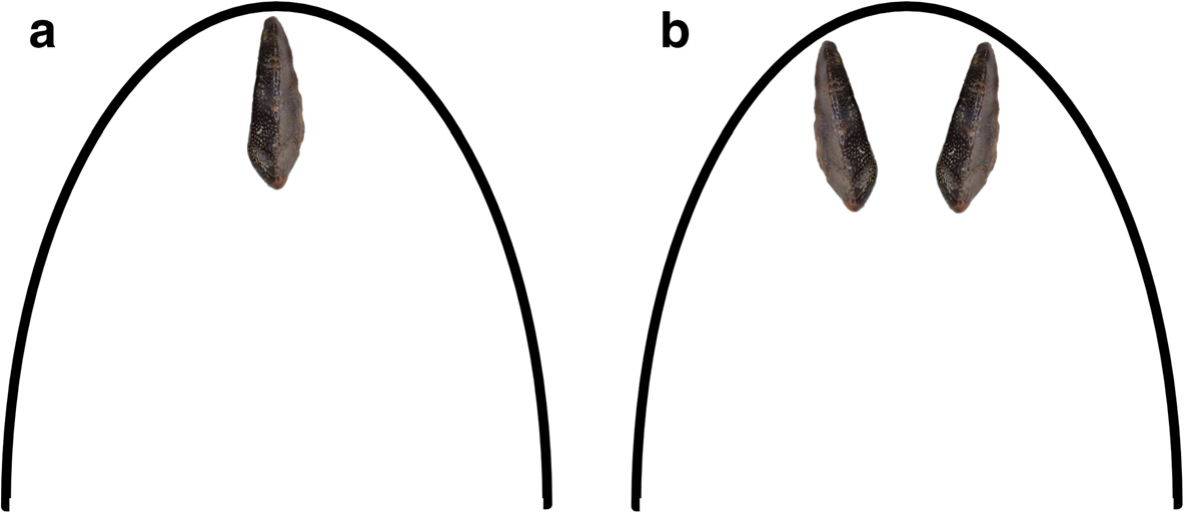
Position of *Arcuodus multicuspidatus* tooth plate if (**a)** symphyseal, (**b)** parasymphyseal

Anterior dentitions in holocephalians are poorly known and not easy to recognize when found isolated. The near-perfect bilateral symmetry of the holotype of *A. multicuspidatus* suggests that it occupied a symphyseal (Fig. 11a) or parasymphyseal (Fig. 11b) position at the anterior end of the jaw. Figure 11a and b show the tooth plate oriented with the wider end lingual and the narrow end labial. This is the orientation that is to be expected if growth is at the lingual end (e.g., lyodont growth) as has been established for other tooth plates [33].

Symphyseal tooth plates are unknown in extant, Cenozoic, or Mesozoic chimaeroids. A symphyseal tooth plate is present in the Mesozoic holocephalian *Myriacanthus paradoxus* [2]. As has already been noted, *H. volsellorhinus* has a symphyseal tooth plate. In cases where the tooth plates appear to be bilaterally symmetric, their position can reasonably be inferred to be symphyseal, even when they are found isolated. Examples are some specimens of *P. rankinei* (Fig. 3), the holotype of *A. multicuspidatus* (Fig. 9a)*,* and the syntype of *Deltodopsis*? *convexus* (Fig. 10a), here referred to *Arcuodus bialveatus.*

### Function of anterior dentitions

Anterior tooth plates might have served to grasp prey, which would be crushed with the larger posterior tooth plates. The multiple nodes on the occlusal surfaces of the holotype of *A. multicuspidatus* or on the anterior tooth plates of *H. volsellorhinus* may have facilitated such a function. An analogy could be made with the extant shark *Heterodontus,* which has files of small, sharp anterior teeth and large, blunt posterior teeth.

### Origin of tooth plates from tooth files

**Fig. 12:**
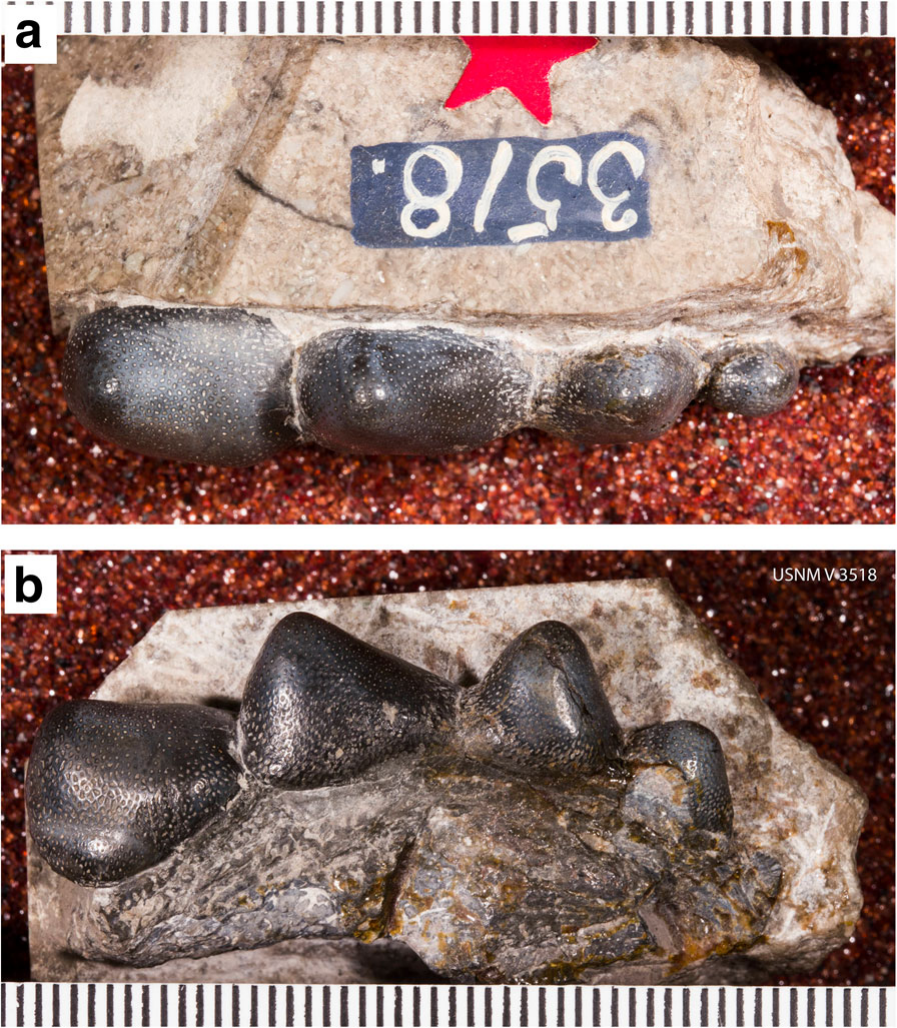
Holotype of *Helodus coxanus* Newberry, 1897, USNM V3518. **a** Occlusal view. Lingual end to left. Millimeter scale divisions. **b** Lateral view. Lingual end to left. Millimeter scale divisions. A drawing of this specimen was published as Newberry, 1897 ([34], pl. 24, fig. 24). Images copyrighted, Smithsonian Institution, all rights reserved

With regard to the evolution of tooth plates from tooth files, it is interesting to compare the tooth whorl that is the holotype of *Helodus coxanus* (Fig. 12) with *A. multicuspidatus.* In *H. coxanus,* the crowns are separate, although the bases appear to be fused. Being bilaterally symmetric, it has always been presumed to have occupied a symphyseal position [34]. The multiple pointed crowns could have been used to grasp prey, similarly to the anterior tooth files of *Heterodontus.* It seems probable that *A. multicuspidatus* was descended from an ancestor having a tooth whorl like that of *H. coxanus.* This does not imply actual descent of *A. multicuspidatus* from *H. coxanus*, nor of *A. bialveatus* from *A. multicuspidatus.* The tooth file of *Helodus coxanus* and the tooth plates of *A. multicuspidatus* and of *A. bialveatus* form a morphological series, but, given the present state of knowledge, it is impossible to know whether or not they form a phylogenetic series. The transition from tooth files to tooth plates very likely occurred independently in several different lineages. The transition from tooth files to tooth plates within the Helodontiformes (e.g., from *Helodus simplex* to *Pleuroplax rankinei*) was most likely independent of the transition, probably within the Cochliodontiformes, that resulted in the tooth plate of *Arcuodus multicuspidatus*.

*Helodus coxanus* was chosen for the comparison because, compared to other, broadly similar tooth files, such as that of *Helodus simplex* (Fig. 2) or of *Helodus appendiculatus* (e.g., NHMUK PV P2916)*,* the relative dimensions of the tooth file are similar to those of the tooth plates here included in *Arcuodus*. The crowns of both *H. simplex* and *H. appendiculatus* are much wider mesio-distally than linguo-labially. Those of *H. coxanus* are much more compressed mesio-distally, so that the overall shape of the tooth file matches that of the tooth plate of *A. multicuspidatus* rather closely. The comparison of *A. multicuspidatus* and *A. bialveatus* is natural, since the two taxa are so close morphologically that they have been assigned to the same genus.

The generic assignment of *Helodus coxanus* deserves some comment. *Helodus simplex* is the type species of *Helodus* and is also known from articulated specimens [19]. All other species of *Helodus* are founded on isolated teeth, many of which probably should be referred to other genera. Many, if not most, of these are anterior teeth of other chondrichthyan fishes, which are known from other remains, such as tooth plates. Until articulated remains are found, there seems to be no way to determine which teeth and tooth plates belong to the same species. Unlike the situation for *A. multicuspidatus* and *A. bialveatus*, no close relationship between *H. coxanus* and *H. simplex* is implied by the fact that they are currently assigned the same genus name.

Table 1 summarizes information regarding taxonomy, age, and morphology for the most important specimens discussed here. Since the ages are for individual specimens, the age ranges for the taxa are unknown, and chondrichthyan species can have rather long age ranges. Because the classifications of the listed taxa are imprecise, the conclusions that can be made as to evolutionary trends are very limited.

**Table 1:** Key specimens with taxonomy, ages, and features

Specimen	Fig.	Original designation	Current designation	Taxonomic group	Age	Features
NHMUK PV P8216	2	*Helodus simplex*	*Helodus simplex*	Helodontidae	Pennsylvanian	Tooth file, teeth mesiodistallly expanded and bilaterally symmetric or nearly so
NHMUK PV P1415	3	*Pleurodus rankinii*	*Pleuroplax rankinei*	Helodontidae	Pennsylvanian	Tooth plate with crenulated edges, bilaterally symmetric
USNM V3518	12	*Helodus coxanus*	*Helodus coxanus*	Cochliodontiformes?	Mississippian (early Viséan)	Tooth file, crowns triangular, mesiodistally compressed, bases fused, bilaterally symmetric
ALMNH PV 2016.0002.0002	9	*Arcuodus multicuspidatus*	*Arcuodus multicuspidatus*	Cochliodontiformes	Mississippian (late Serpukhovian)	Tooth plate with sharp median ridge, sharp cusps, bilaterally symmetric or nearly so
USNM V13017	6	*Deltodopsis*? *bialveatus*	*Arcuodus bialveatus*	Cochliodontiformes	Mississippian (late Tournasian)	Tooth plate with low, irregular bulges, not bilaterally symmetric
AMNH FF6450	7	*Deltodopsis*? *bialveatus*	*Arcuodus bialveatus*	Cochliodontiformes	Mississippian (early Viséan)	Tooth plate with low, wide, transverse ridges, not bilaterally symmetric
USNM V13015	10	*Deltodopsis*? *convexus*	*Arcuodus bialveatus*	Cochliodontiformes	Mississippian (late Tournasian)	Tooth plate with very low surface undulations, bilaterally symmetric or nearly so

## Conclusions

The new tooth plate from the Bangor Limestone of Alabama, USA, is referred to a new genus and species, *Arcuodus multicuspidatus*. The holotype and only known specimen is interpreted as having occupied an anterior position. The multicusped morphology of the tooth plate suggests that it might have had a grasping function. The multiple cusps suggest that *A. multicuspidatus* might have evolved from a fish having a tooth file like that of *Helodus coxanus*, having separate teeth. *A. bialveatus*, which possesses a tooth plate that lacks prominent cusps, may have evolved from a fish possessing a tooth plate similar to that of *A*. *multicuspidatus*. The age of *A. multicuspidatus* is middle to latest Chesterian (Serpukhovian). Several other tooth plates questionably referred to *Deltodopsis* by St. John and Worthen are referred to *Arcuodus bialveatus* comb. nov. The dentitions of the three taxa: *Helodus coxanus*, *Arcuodus multicuspidatus*, and *Arcuodus bialveatus* form a morphological sequence. Whether they also form a phylogenetic sequence is not possible to determine with the present evidence.

## Abbreviations

ALMNH: Alabama Museum of Natural History, Tuscaloosa, AL, USA
AMNH: American Museum of Natural History, New York, NY, USA
NHMUK: Natural History Museum, London, UK
USNM: National Museum of Natural History, Washington, DC, USA
